# Supplementary material for: CRISPR-FRT targets shared sites in a knock-out collection for off-the-shelf genome editing
Source: Nat Commun. 2018 Jun 8;9:2231. doi: 10.1038/s41467-018-04651-5 (PMC5993718; doi:10.1038/s41467-018-04651-5)
Supplement: Supplementary file 1 — Supplementary Information [file 41467_2018_4651_MOESM1_ESM.pdf]

CRISPR-FRT targets shared sites in a knock-out collection for off-the-shelf genome editing

Toon Swings[§], David C. Marciano[§], Benu Atri, Rachel E. Bosserman, Chen Wang, Marlies Leysen, Camille Bonte, Thomas Schalck, Ian Furey, Bram Van den Bergh, Natalie Verstraeten, Peter J. Christie, Christophe Herman, Olivier Lichtarge[*], Jan Michiels[*]

Supplementary Information

This file contains: - 2 Supplementary figures

Supplementary figures

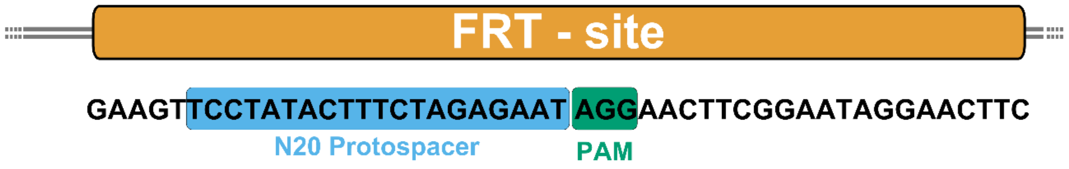

Supplementary Figure 1: Location of the protospacer N20 sequence and selection of the PAM-site to target with Cas9. In the 48 nt-long FRT sequence multiple PAM-sites (-NGG) are available. The best performing PAM site was determined using an online available gRNA design tool (see **Methods**) for *E. coli* K12.

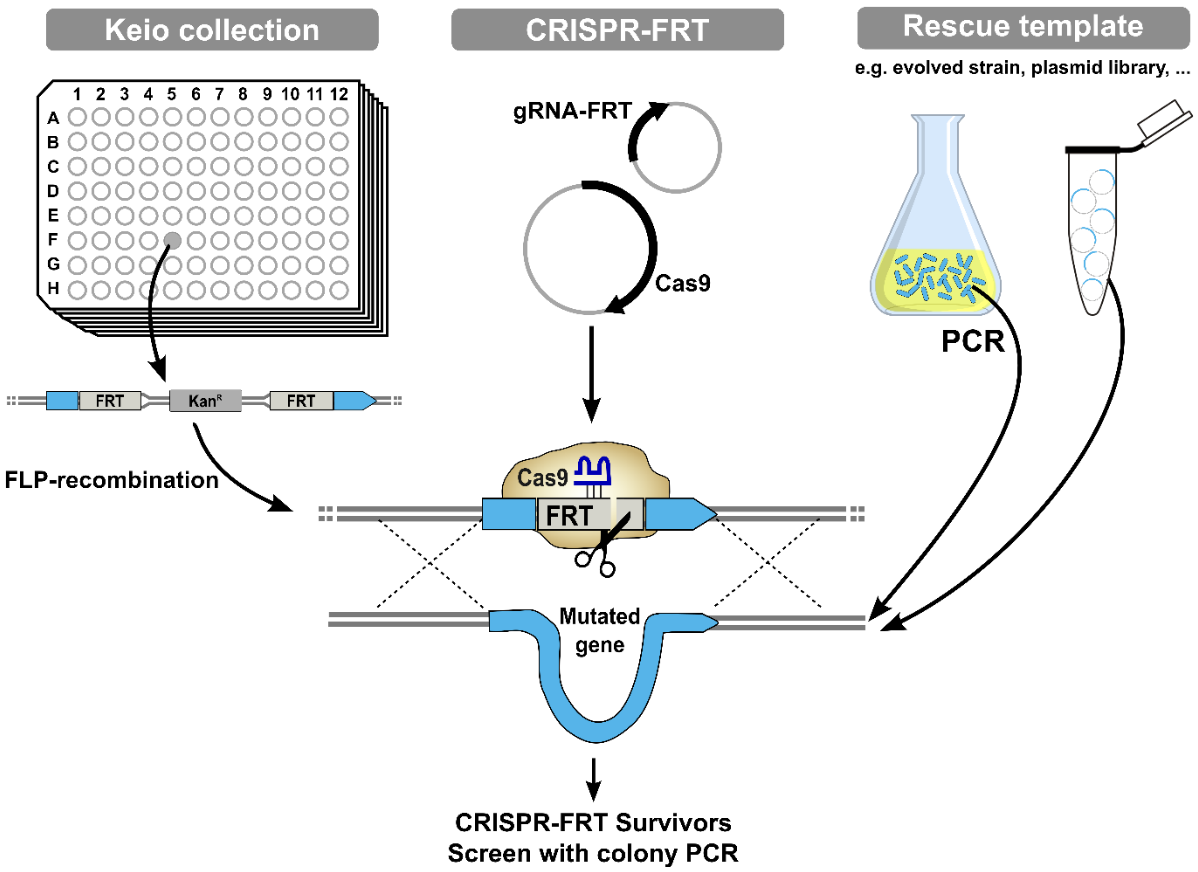

Supplementary Figure 2: **Overview of the CRISPR-FRT protocol if targeting a single FRT site**. To use CRISPR-FRT when the Cas9 or gRNA delivery vector or the library plasmids already harbors a Kan^R cassette, first the Kan^R cassette from the Keio mutant needs to be removed by FLP recombination (see **methods**). Next, the single remaining FRT-site can be targeted by Cas9 guided by the gRNA-FRT. A convenient rescue template (e.g. a mutated gene from an evolved *E. coli* strain amplified by PCR, a plasmid-encoded gene variant, etc.) recombines (dashed lines) over the homologous regions flanking the FRT-site. Survivors are screened by colony PCR with primers flanking the FRT-site.
