# Supplementary material for: CRISPR-FRT targets shared sites in a knock-out collection for off-the-shelf genome editing
Source: Nat Commun. 2018 Jun 8;9:2231. doi: 10.1038/s41467-018-04651-5 (PMC5993718; doi:10.1038/s41467-018-04651-5)
Supplement: Supplementary file 3 — Description of Additional Supplementary Files [file 41467_2018_4651_MOESM3_ESM.pdf]

Description of Additional Supplementary Files

File Name: Supplementary Data 1
Description: This table contains all the strains and plasmids used or constructed in this study.

File Name: Supplementary Data 2
Description: This table contains all primer sequences used in this study.
